# The six-transmembrane protein Stamp2 ameliorates pulmonary vascular remodeling and pulmonary hypertension in mice

**DOI:** 10.1007/s00395-020-00826-8

**Published:** 2020-11-13

**Authors:** Mehreen Batool, Eva M. Berghausen, Mario Zierden, Marius Vantler, Ralph T. Schermuly, Stephan Baldus, Stephan Rosenkranz, Henrik ten Freyhaus

**Affiliations:** 1 Cologne Cardiovascular Research Center (CCRC), and Center for Molecular Medicine Cologne (CMMC), Klinik III Für Innere Medizin, Herzzentrum Der Universität Zu Köln, Kerpener Str. 62, 50937 Köln, Germany; 2 Universities of Giessen and Marburg Lung Center (UGMLC), German Center for Lung Research (DZL), Giessen, Germany; 3 German Center for Lung Research (DZL), Giessen, Germany

**Keywords:** Pulmonary hypertension, Inflammation, Stamp2, Vascular remodeling, Macrophages

## Abstract

Six-transmembrane protein of prostate (Stamp2) protects from diabetes and atherosclerosis in mice via anti-inflammatory mechanisms. As chronic inflammation is a hallmark of pulmonary arterial hypertension (PAH), we investigated the role of Stamp2. Stamp2 expression was substantially reduced in the lung of humans with idiopathic PAH, as well as in experimental PAH. In Stamp2-deficient mice, hypoxia modestly aggravated pulmonary vascular remodeling and right ventricular pressure compared to WT. As endothelial cell (EC) and pulmonary arterial smooth muscle cell (PASMC) phenotypes drive remodeling in PAH, we explored the role of Stamp2. Knock-down of Stamp2 in human EC neither affected apoptosis, viability, nor release of IL-6. Moreover, Stamp2 deficiency in primary PASMC did not alter mitogenic or migratory properties. As Stamp2 deficiency augmented expression of inflammatory cytokines and numbers of CD68-positive cells in the lung, actions of Stamp2 in macrophages may drive vascular remodeling. Thus, PASMC responses were assessed following treatment with conditioned media of primary Stamp2^−/−^ or WT macrophages. Stamp2^−/−^ supernatants induced PASMC proliferation and migration stronger compared to WT. A cytokine array revealed CXCL12, MCP-1 and IL-6 as most relevant candidates. Experiments with neutralizing antibodies confirmed the role of these cytokines in driving Stamp2’s responses. In conclusion, Stamp2 deficiency aggravates pulmonary vascular remodeling via cross-talk between macrophages and PASMC. Despite a substantial pro-inflammatory response, the hemodynamic effect of Stamp2 deficiency is modest suggesting that additional mechanisms apart from inflammation are necessary to induce severe PAH.

**Electronic supplementary material:**

The online version of this article (10.1007/s00395-020-00826-8) contains supplementary material, which is available to authorized users.

## Introduction

Pulmonary arterial hypertension (PAH) is characterized by chronic elevation of pulmonary arterial pressure and pulmonary vascular resistance, affecting approximately 100 million people worldwide [2, 25]. If left untreated, historical data from PAH patients obtained in the 1980s demonstrate a median survival of only 2.8 years [10], predominantly due to right ventricular failure [14, 46]. The development and approval of targeted therapies led to improvement of survival, morbidity events and quality of life. Nevertheless, annual mortality is still unacceptably high [5, 18, 28]. Because disease-modifying drugs directly targeting the vascular remodeling process in PAH are still lacking, there is an urgent need for novel concepts and therapeutic targets.

On the cellular level, dysregulation and apoptosis of endothelial cells and smooth muscle cell responses drive vascular remodeling processes in PAH [12]. Furthermore, inflammatory processes are linked to disease progression [8, 16, 20], and plasma levels of cyto- and chemokines such as interleukin (IL)-1β, IL-6, monocyte chemotactic protein (MCP)-1 and tumor necrosis factor (TNF)-α have prognostic value [3, 21, 39, 42]). A potential source of increased circulating mediators are perivascular infiltrates of mononuclear cells that were described in the lung of PAH patients [9, 29]. However, the molecular connection(s) between inflammation and vascular remodeling remain to be deciphered. A potential explanation arises from the notion that secreted factors from leukocytes have mitogenic, chemotactic and vasoconstrictive effects, including IL-6 [37], platelet-derived growth factor (PDGF) [38, 45] and endothelin (ET)-1 [13]. Many of these factors are secreted by macrophages that are causally linked to PAH disease progression [26, 48].

Stamp2 is a member of the Stamp or Steap (Six-Transmembrane Epithelial Antigen of Prostate) family of 6-transmembrane proteins. Three of the four family members (Stamp1 (also termed Steap2), Stamp2 (also Steap4 or TNF-induced adipose-related protein) and Steap3) share an N-terminal NADP^+^-oxidoreductase domain [34] with homology to the F_420_H_2_:NADP^+^-oxidoreductase of archaea and bacteria [19, 31, 36]. Due to cytoplasmic orientation, the domain works as an electron donor, mediating the reduction of iron and copper [33, 34]. Stamp2 is expressed in adipose tissue, and normal upregulation of the protein by nutrient uptake is defective in obese models. Stamp2 deficiency in mice led to inflammation of visceral adipose tissue and to metabolic syndrome [51]. Thus, Stamp2 appears to play an important role in adipose tissue through its ability to integrate metabolic and inflammatory responses. In mouse models of inflammation [22, 27], as well as in human adipose tissue, Stamp2 expression at least in part co-localizes with the macrophage marker CD68 [1]. We [44] and others [17] highlighted the role of Stamp2 in macrophages. In these cells, Stamp2 controls NADPH homeostasis and NF-κB-dependent secretion of inflammatory cytokines (IL-6, IL-1β, TNF-α, and MCP-1) via the N-terminal oxidoreductase domain. As a consequence, lack of Stamp2 expression led to early atherosclerosis in ApoE^−/−^-deficient mice. Bone marrow transplantation experiments revealed that leukocytes are critical for the observed phenotype.

Collectively, these data demonstrate that Stamp2 is a critical anti-inflammatory protein in macrophages that has a role in vivo in atherogenesis. Here we report that Stamp2 deficiency in mice aggravates hypoxia-induced pulmonary vascular remodeling and PAH via actions in macrophages and their cross-talk with vascular smooth muscle cells. Stamp2 expression is decreased in human and experimental PAH suggesting that loss of this protective factor is implicated in disease progression.

## Experimental procedures

### Cells, cell culture and exposure to hypoxia in vitro

Human PASMC (lots 00003639143 (age: 43y, sex: m, race: c) and 13,981 (age: 2,5 m, sex: m, race: c) and human microvascular endothelial cells (MVEC, lots 0000489936 (age: 2y, sex: f, race: b), 0000580578 (age: 57y, sex: m, race: h) and 0000582655 (age: 17y, sex: m, race: h) were obtained from Lonza (Basel, Switzerland), and were maintained in the manufacturers “Clonetics SmGm” (Smooth Muscle cell Growth Medium) or “Clonetics EBM” (Endothelial cell Basal Medium with Supplements). Mouse PASMC were isolated from either wildtype (WT) or Stamp2-deficient mice as follows. After sacrifice, the pulmonary artery was carefully dissected. The adventitia was removed using anatomic forceps and the endothelium was gently removed by scraping the luminal surface. The pulmonary artery was cut into pieces and incubated in an enzyme solution (collagenase, elastase, trypsin-inhibitor) at 37 °C for 90 min to disintegrate the tissue. PASMCs were separated by centrifugation (420×*g*, 2 min) and then resuspended in DMEM culture medium containing 20% FCS and 1% Penicillin/Streptomycin. After reaching 80% confluence, cells were expanded. Experiments were performed with cells from passages 5–12. Cells were grown under normoxic conditions (5% CO2, 95% air, 37 °C) in a water-jacketed incubator or exposed to hypoxic conditions (1% O2, 5% CO2, 37 °C) in modular incubation chambers (Billups-Rothenberg).

For the isolation of peritoneal macrophages, mice were injected intraperitoneally with thioglycollate 3 days prior to harvesting of cells. Mice were sacrificed and 10 ml of cold PBS was injected into the peritoneal cavity and reabsorbed. After centrifugation (150×*g*, 10 min) the pelleted cells were resuspended in DMEM. After 2 h, attached macrophages were transferred to culture dishes.

### Human tissue samples

The study protocol for tissue donation was approved by the Ethics Committee of the University of Giessen (Giessen, Germany) in accordance with national law and with the principles of the Declaration of Helsinki. Written informed consent was obtained prior to inclusion. Human lung tissues were obtained from individuals with IPAH undergoing lung transplantation or from non-transplanted donor lungs. Explanted lungs were either snap-frozen or fixed in 4% phosphate-buffered paraformaldehyde (Santa Cruz Biotechnology, Santa Cruz, USA), dehydrated and paraffin embedded, and 3 μm sections were obtained.

### Animals and animal models of pulmonary hypertension

Stamp2-deficient mice were kindly provided by Prof. Gökhan S. Hotamisligil (Sabri Ülker Center, Department of Molecular Metabolism and Broad Institute of Harvard-MIT and Harvard T.H. Chan School of Public Health, Boston, US). Handling and breeding of the animals and all experimental procedures were performed in accordance to the German Laws for Animal Protection and conform to the guidelines from Directive 2010/63/EU of the European Parliament. They were approved by the local animal care committee and district government of Cologne (84-02.04.2013.A108, 4.18.004).

*Hypoxia-induced pulmonary hypertension* Male Stamp2-deficient (Stamp2^−/−^) and WT mice (Stamp2^+/+^) aged between 8–14 weeks were exposed to a normobaric (10% oxygen) hypoxia for 21 days or to normoxia, respectively.

*Sugen/hypoxia* (SuHx)-induced pulmonary hypertension [7]: male Sprague Dawley rats (8 weeks of age) were subcutaneously injected with Sugen 5416 (20 mg/kg dissolved in DMSO) followed by exposure to hypoxia for 3 weeks and to normoxia for another 2 weeks.

### Hemodynamic measurements

Hemodynamic analyses were performed after 21 days of hypoxia/normoxia exposure. Anesthesia and analgesia were realized through isoflurane (2%) and carprofen (5 mg/kg body weight, subcutaneously injected 30 min before start of the operation). The operation was started when pain stimuli were no longer perceptible (failure of the inter-toe reflex). Right ventricular systolic pressure (RVSP) was measured utilizing a Millar microtip pressure catheter inserted into the right ventricle via the jugular vein. Systemic arterial blood pressure (SAP) was monitored in the contralateral carotid artery. The catheter information was amplified by a PowerLab® amplifier and converted to pressure curves using LabChart7® software (AD instruments, Sydney, Australia). Euthanasia was realized through an isoflurane overdose (7%) and subsequent intracardiac puncture and blood sampling of approximately 1 ml.

### Assessment of right ventricular hypertrophy

To assess right ventricular (RV) hypertrophy, the RV was dissected from the left ventricle (LV) including ventricular septum, and wet weight was obtained separately. Right ventricular hypertrophy is demonstrated as an increased RV to LV (free wall and ventricular septum) weight ratio (RV/LV + S).

### Tissue preparation

Lungs were perfused with PBS for 5–10 min and either snap-frozen or fixed in 4% phosphate-buffered paraformaldehyde. Following dehydration, lungs were embedded in paraffin and sectioned into 3 µm sections for immunohistochemistry.

### Immunohistochemistry

Immunohistochemistry was performed using antibodies directed against Stamp2 (Steap4 #11944-1, Proteintech, Rosemont, USA), CD68 (#137001, BioLegend, San Diego, USA), von Willebrand factor (A0082, Dako, Santa Clara, USA), and α-smooth muscle actin (A2547, Sigma-Aldrich, St. Lousis, USA). Secondary antibody for CD68 and a-smooth muscle actin was a mouse-on-mouse HRP Polymer Kit (Zytomed systems, Bargteheide, Germany). For Stamp2 and von Willebrand factor, the ImmPress horse anti-rabbit polymer peroxidase kit (Vector laboratories, Inc., Burlingame, USA) was used. Negative controls were performed by omission of the primary antibody. Specificity of Stamp2 antibody is demonstrated in Supplementary Fig. 1.

**Fig. 1:**
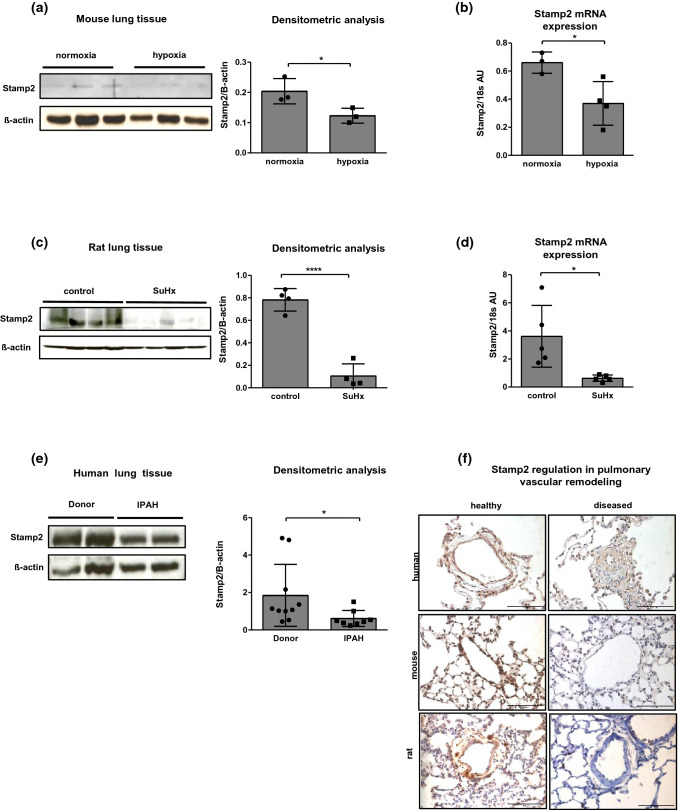
Stamp2 expression is reduced in human and experimental PAH. **a** Immunoblot and densitometric analyses, demonstrating Stamp2 expression in lung tissue from hypoxia-challenged mice compared to normoxic control mice (*n* = 3,3). **b** Stamp2 mRNA expression in lung tissue of the above mentioned mice (*n* = 3,4,4). **c** Stamp2 protein expression and densitometric analysis (*n* = 4,4) and **d** mRNA expression in lung tissue of Sugen5416/hypoxia (SuHx)-treated rats compared to healthy control rats (*n* = 5,5). **e** Stamp2 expression in lung tissue from IPAH patients as compared to healthy donors (*n* = 10,8). **f** Immunohistochemical stainings demonstrating reduced Stamp2 expression in pulmonary vessels from IPAH patients and from experimental PAH versus controls (400 × magnification). All data represent means ± SD.*p < 0.05, ****p < 0.0001 as assessed by two-tailed students *t*-test

### Quantification of vascular muscularization and of CD68^+^ macrophages

α-Actin and von Willebrand factor (vWF) double-immunostained lung tissue sections were analyzed in a 400 × magnification using Keyence BZ II analyzer software. Total vessel, vascular lumen and α-actin-positive areas were manually labeled and the vascular wall area was calculated. Measurement of the vessel diameter and vascular wall area were used to draw the percentage of muscularized area in proportion to the vessel area. The degree of muscularization was entitled as non-muscularized (< 5% α-actin stained area), as partially muscularized (5–69% stained area) or as fully muscularized (> 70% stained area). In each tissue section, 50–80 intra-acinar arteries were analyzed.

For the quantification of CD68^+^ infiltrates a number of ten vessels out of 5 lungs for each condition were analyzed. Quantification is expressed as the percentage of CD68^+^ vessels to the total of 10 vessels (60-fold magnification).

### siRNA transfection

Stamp2 siRNA (target sequence: 5′-CACAATGGTGACCACTGATAA-3′, sense strand: 5′-CAAUGGUGACCACUGAUAATT-3′, antisense strand: 5′-UUAUCAGUGGUCAUUGTG-3′, Qiagen, Hilden, Germany) or non-silencing siRNA were transfected into the cells using INTERFERin® transfection reagent according to the manufacturer’s protocol (Polyplus, New York City, USA). Sufficient down-regulation was achieved after 48 h.

### BrdU incorporation

DNA synthesis as a measure of cellular proliferation was obtained by a 5-bromodeoxyuridine (BrdU)-incorporation assay (Cell Proliferation ELISA, Roche Diagnostics, Rotkreuz, Switzerland). Cells were cultured in 96-well plates (1 × 10^4^ cells per well) in the appropriate medium. After 24 h of serum-deprivation, cells were incubated with the indicated factors for 24 h. BrdU incorporation was carried out according to the manufacturer´s specifications. Quantification was performed by measuring the absorbance at 370 and 492 nm using a Power Wave 340 ELISA reader (Bio-TEK Instruments, Winooski, USA).

### Scratch assay

PASMC were plated in 24-well plates at a density of 1 × 10^5^ cells per well. After 24 h, cells were serum-deprived for 24 h. Using a 1000 µl tip, a straight scratch was made vertically in the center of the well, and the cells were incubated with the indicated agents. The level of scratch cover was determined after 24 h using ImageJ software and is displayed relative to the area of the scratch without treatment as the relative scratch gap.

### Apoptosis

Apoptosis was determined using a Cell Death Detection ELISA assay (Roche Diagnostics, Rotkreuz, Switzerland), based on the detection of cytoplasmic histone-associated DNA fragments in apoptotic cells. Cells were plated in 96-well plates in the appropriate medium (1 × 10^4^ cells per well). After 24 h the cells were serum-deprived for 24 h followed by incubation with 100 µM hydrogen peroxide for 24 h. After fixation of cell lysates, the amount of cell death was quantified using the Cell Death Detection ELISA assay according to the manufacturer´s specifications. Quantification was performed by measuring the absorbance at 405 and 490 nm using a Power Wave 340 ELISA reader (Bio-TEK Instruments, Winooski, USA).

### Viability assay

The effect of Stamp2 deficiency on cellular survival was assessed by a 3-(4,5-dimethylthiazol-2-yl)-2,5-diphenyltetrazolium bromide (MTT) assay (Sigma-Aldrich, St. Lousis, USA). Cells were cultured in 96-well plates (1 × 10^4^ cells per well) in the appropriate medium (Clonetics EBM, Lonza, Basel, Switzerland). Optical density was determined at a wavelength of 600 nm after 4 h of incubation.

### Cytokines and neutralizing antibodies

Recombinant human IL-6 (#AF-200-06, PeproTech, Rocky Hill, USA), recombinant murine SDF-1a (CXCL12) (#250-20A, PeproTech, Rocky Hill, USA), recombinant murine MCP-1 (#250-10, PeproTech, Rocky Hill, USA), Anti-hIL-6-IgG (#mabg-hil6-3, InvitroGen, Carlsbad, USA), Anti-SDF-1 (CXCL12) antibody (ab9797, Abcam, Cambridge, UK), Anti-MCP-1 antibody (ab25124, Abcam, Cambridge, UK).

### Protein extraction and immunoblotting

The lung tissue was homogenized using RIPA lysis buffer (Merck, Darmstadt, Germany). Cells were lysed in EB (10 mM Tris–HCl, pH 7.4, 5 mM EDTA, 50 mM NaCl, 50 mM NaF, 1% Triton X-100, 0.1% bovine serum albumin, 20 μg/ml aprotinin, 2 mM Na_3_VO_4_, 1 mm phenylmethylsulfonyl fluoride). Lysates were centrifuged (20 min, 12,000×*g*). Protein concentration of the supernatants was assessed by either Bradford Assay (Bio-Rad) or Nanodrop quantification. Homogenates were re-suspended in 4 × SDS sample buffer. Equal amounts of protein lysates were run on SDS–PAGE and transferred to PVDF membranes. Blots were probed with the indicated antibodies. The protein detection was carried out by using the Pierce ECL Western Blotting Substrate kit (Thermo Fisher Scientific, Waltham, USA). Under light exposure, photosensitive X-ray films (GE Healthcare AmershamTM Hyperfilm ECL, Fisher Scientific, Waltham, USA) were exposed to the membranes and developed via Curix 60 developer (AGFA-Gevaert, Cologne, Germany). Protein bands on developed films were scanned and the image was analyzed using Photoshop software (Adobe). Intensity of individual bands for each sample was calculated, background noise was subtracted and all values were normalized to their respective control bands.

### QRT PCR

RNA isolation was performed using RNeasy Mini Kit (Qiagen, Hilden, Germany) according to the manufacturer’s specifications. cDNA was synthesized using SuperScript™ Kit (Invitrogen, Carlsbad, USA) according to the manufacturer’s instructions. SYBRGreen—based (Thermo Fisher Scientific, Waltham, USA) qPCR of various genes (TNF-α Forward: CCACCACGCTCTTCTGTCTAC, Reverse: TCCGAGGTCCTGACTCTGTC; MCP-1 Forward: CCACTCACCTGCTGCTACTAC, Reverse: TGGTGATCCTCTTGTAGCTCTCC; IL-1β Forward: ATCTTTTGGGGTCCGTCAACT, Reverse: CACGATTTCCCAGAGAACATGTG; IL-6 Forward: ACAACCACGGCCTTCCCTACT, Reverse: CACGATTTCCCAGAGAAC; Stamp2 Forward: TCAAATGCGGAATACCTTGCT, Reverse: GCATCTAGTGTTCCTGACTGGA; Human IL-6 Forward: CCAGCTATGAACTCCTTCTC, Reverse: GCTTGTTCCTCACATCTCTC; Human Stamp2 Forward: CAGAGTACCTTGCTCATTTGGT, Reverse: TGTCATTTCCACACACAAACAC; 18S Forward: AGTCCCTGCCCTTTGTAC, Reverse: CGATCCAGGGCCTCACTA) was performed on a LightCycler® 480 instrument (Roche Diagnostics, Rotkreuz, Switzerland). Gene expression is shown in relation to the housekeeping gene 18S rRNA expression.

### Cytokine array

Cytokine and chemokine expression in macrophage supernatants from WT and Stamp2-deficient mice were analyzed using Mouse Cytokine array panel A (R&D systems, Minneapolis, USA) according to the manufacturer’s instruction. Selected capture antibodies spotted in duplicates on nitrocellulose membranes were incubated with the supernatant mixed with biotinylated detection antibody mix according to the provided instructions. The cytokine/detection antibody complexes were bound by its cognate immobilized capture antibody on the membrane. Streptavidin–Horseradish Peroxidase and chemiluminescent detection reagents were added, and a signal is produced in proportion to the amount of cytokine bound. Chemiluminescence was detected using photosensitive X-ray films (GE Healthcare AmershamTM Hyperfilm ECL, Fisher Scientific, Waltham, USA).

### Immunofluorescence

Tissue sections were deparaffinized and rehydrated. Antigen retrieval was performed using 1% Triton X in PBS for 10 min. Immunofluorescence staining was performed by incubation with a phospho-specific p65 antibody (#3033, cell signaling, 1:50) overnight. Slides were subsequently washed and incubated with an Alexa 488-conjugated secondary antibody (#4412, Cell Signaling, Cambridge, UK, 1:100), counterstained with nuclear DAPI (1 µg/ml, #10236276001, Sigma-Aldrich, St. Louis, USA) and mounted with fluorescent mounting medium (#S3023, Dako, Santa Clara, USA).

### Statistical analyses and graphics

All data are expressed as mean values ± SD obtained from at least three independent experiments. Statistical analyses were performed using Student´s *t*-test or ANOVA (analysis of variance) as indicated. Statistical significance was defined as *p < 0.05, **p < 0.01, ***p < 0.001, ****p < 0.0001. Graphics were created and statistics were calculated with Graphpad prism version number 5.03.

## Results

### Stamp2 is downregulated in experimental and human PAH

We analyzed the expression of Stamp2 in two distinct experimental models of PAH (the murine hypoxia-model and the model of severe PAH, sugen/hypoxia in rats) and in human idiopathic pulmonary arterial hypertension (IPAH). Immunoblot analyses of lung tissue homogenates from hypoxia-challenged mice demonstrated strongly reduced levels of Stamp2 as compared to normoxia (*p* < 0.05) (Fig. 1a). In line with this observation, Stamp2 transcript levels were also reduced under hypoxic conditions (*p* < 0.05) (Fig. 1b). Furthermore, in the SuHx rat model of severe PAH, a profound downregulation of Stamp2 protein was found as compared to healthy controls (*p* < 0.0001) (Fig. 1c), which was also corroborated by downregulation of Stamp2 transcript levels by qPCR (*p* < 0.05) (Fig. 1d.). The finding of reduced Stamp2 expression in PAH was not confined to animal models, as shown by analyses of lung samples from patients with IPAH by immunoblotting and immunohistochemistry (Fig. 1e,f). Furthermore, Stamp2 expression appeared to be localized mainly to the pulmonary vessels itself (Fig. 1f).

### Lack of Stamp2 augments hypoxia-induced vascular remodeling and PAH in mice

**Fig. 2:**
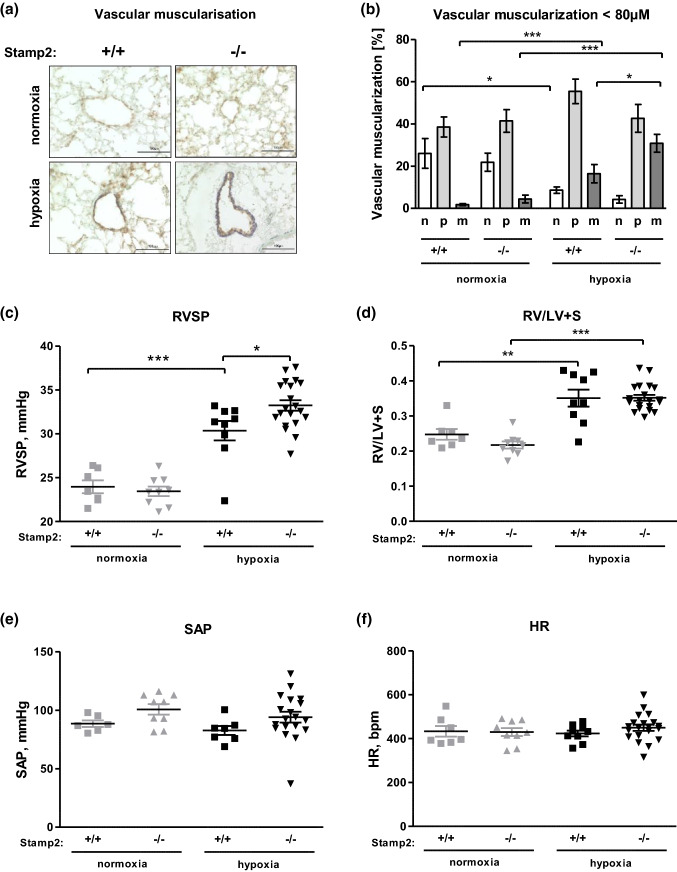
Lack of Stamp2 augments hypoxia-induced PAH in mice. Pulmonary and systemic hemodynamics and pulmonary vascular remodeling of WT versus Stamp2-deficient mice exposed to hypoxia (10% O_2_ for 21 days). **a** Immunohistochemical stainings of small pulmonary arteries from Stamp2^−/−^ and WT mice. Shown are representative images of lung sections immunostained for von Willebrand factor (vWF) (brown) and α-smooth muscle actin (purple) (400 × magnification). **b** Quantitative morphometric analysis of the muscularization of small (< 80 µm) pulmonary arteries. Shown is the percentage of fully (M), partially (P) and non-muscularized (N) vessels (analyses of at least 50/animal, *n* = 4,5,5,5). **c** Right ventricular systolic pressure (RVSP, [mmHg]), measured by Millar microtip catheters (1F) inserted into the right ventricle via the jugular vein (*n* = 7,9,9,20). **d** Right ventricular (RV) hypertrophy shown as RV/LV + S ratio (*n* = 7,9,9,20). **e** Systemic arterial pressure (SAP, [mmHg]) measured using a Millar microtip catheter (1F), inserted into the left carotid artery (*n* = 6,7,9,19). **f** Heart rate (HR, [bpm]) (*n* = 7,9,9,20). All data represent means ± SD. *p < 0.05, **p < 0.01, ***p < 0,001 as assessed by ANOVA or two-tailed students *t*-test

To test the hypothesis that decreased Stamp2 expression is not a mere bystander but rather causally involved in the development of PAH, Stamp2^+/+^ and Stamp2^−/−^ mice were subjected to the well-established model of chronic hypoxia-induced pulmonary hypertension. In this setting, morphometric analyses of small pulmonary arteries (diameter < 80 μm) were performed to characterize the extent of hypoxia-induced pulmonary vascular remodeling. Lung tissue sections were double-stained for von Willebrand factor (vWF) and for α-smooth muscle actin to visualize the endothelial and the smooth muscle cell layers, respectively (Fig. 2a). As expected, hypoxia led to an increase in the percentage of muscularized pulmonary arteries in Stamp2^+/+^ mice, while the percentage of non-muscularized arteries decreased. These effects were substantially aggravated in Stamp2-deficient mice (*p* < 0.05), demonstrating further augmentation of vascular remodeling in response to hypoxia in the absence of Stamp2 (Fig. 2b). In line with this observation, exposure to chronic hypoxia (10% O_2_ for 3 weeks) also moderately augmented PAH in Stamp2^−/−^ mice, as indicated by a significantly higher increase of the right ventricular systolic pressure (RVSP) to 33.2 ± 0.6 mmHg (*p* < 0.001) as compared to Stamp2^+/+^ mice (30.4 ± 1.1 mmHg; *p* < 0.05) (Fig. 2c). There were no significant alterations of right ventricular hypertrophy, systolic arterial blood pressure (SAP) and heart rate (HR) between genotypes and treatment groups (Fig. 2d–f).

In conclusion, these data indicate that the absence of Stamp2 predisposes to the development of severe hypoxia-induced pulmonary vascular remodeling and PH.

Together with the observation of decreased Stamp2 expression in experimental and human idiopathic PAH, these data suggest that downregulation of Stamp2 is a critical event during development and progression of PAH.

### Hypoxia downregulates Stamp2 expression in cell types related to vascular remodeling processes

**Fig. 3:**
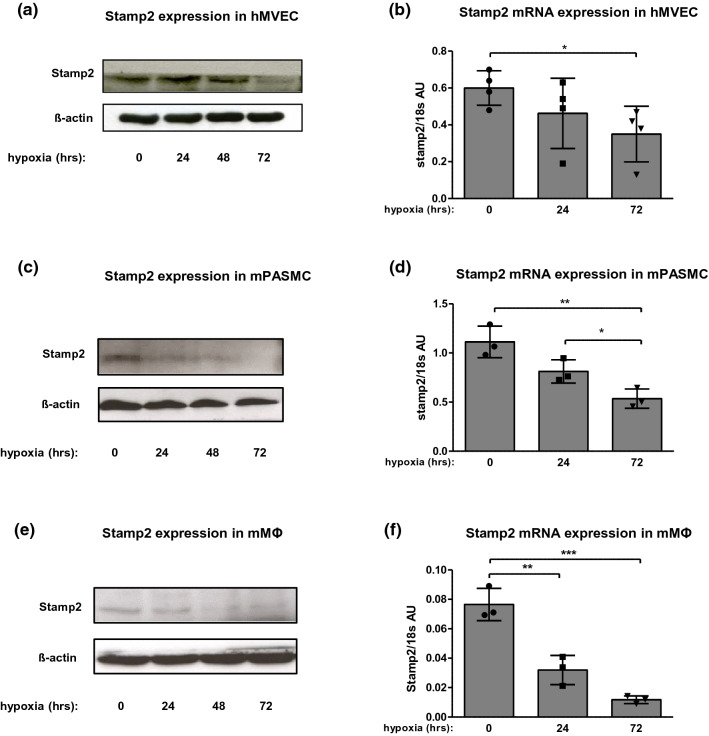
Hypoxia promotes downregulation of Stamp2 expression in various cell types. Expression of Stamp2 **a** protein and **b** mRNA in human MVEC in response to 0, 24, 48, 72 h of hypoxia (1% O_2_) (*n* = 4). Expression of Stamp2 protein (**c**) and mRNA (**d**) in hypoxia-exposed murine PASMC at the indicated time points (*n* = 3). Stamp2 expression protein (**e**) and mRNA (**f**) in murine macrophages (*n* = 3). All data represent means ± SD. *p < 0.05, **p < 0.01, as assessed by two-tailed students *t*-test

Given the finding that Stamp2 expression was detected mainly in or surrounding pulmonary vessels, we evaluated Stamp2 expression in endothelial and smooth muscle cells, as these are critically involved in pulmonary vascular remodeling [47] and furthermore in macrophages. The latter cell type was included due to earlier work defining a role for Stamp2 in macrophages [44] and the established role of this cell type in PAH. Pulmonary microvascular endothelial cells (MVEC), pulmonary arterial smooth muscle cells (PASMC), and macrophages were exposed to hypoxia for 24, 48, and 72 h. In both MVEC and PASMC, Stamp2 protein and mRNA levels were strongly decreased by hypoxia (*p* < 0.05) (Fig. 3a–d). However, the most robust downregulation was detected in macrophages. In these cells, a strong decrease of Stamp2 expression occurred already after 24 h of hypoxia and expression further declined at 48 and 72 h (Fig. 3e–f). Thus, hypoxia leads to Stamp2 downregulation in endothelial cells, smooth muscle cells, and macrophages which are all involved in vascular remodeling during PAH.

### Stamp2 deficiency/downregulation does not affect PASMC and MVEC responses

**Fig. 4:**
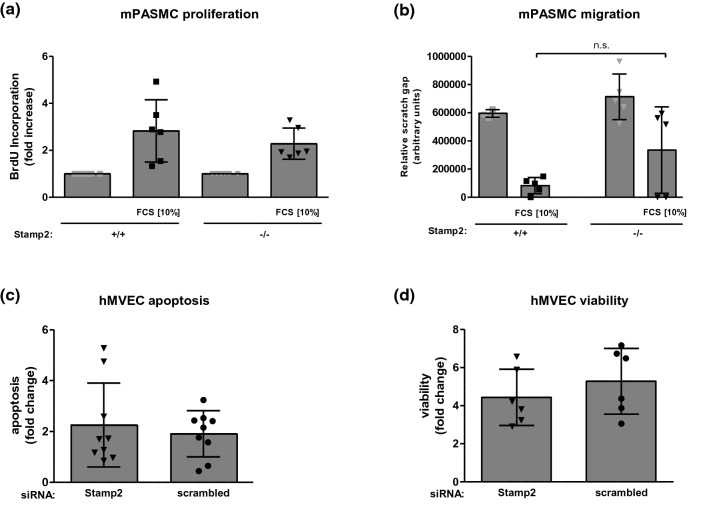
Stamp2 deficiency/downregulation does not affect PASMC and MVEC responses. **a** BrdU incorporation in primary Stamp2-deficient and WT PASMC in response to FCS [10%] and IL-6 [15 ng/ml] (*n* = 6)) and **b** PASMC-migration in response to FCS [10%] (*n* = 5). **c** Apoptosis (*n* = 9) and **d** cellular viability (*n* = 6) of human MVEC transfected with either siRNA targeting Stamp2 or non-silencing siRNA for 48 h. All data represent means ± SD

Since proliferation and migration of smooth muscle cells, as well as apoptosis resistance of endothelial cells represent critical cellular responses relevant to vascular remodeling, we investigated the impact of Stamp2 deficiency on these events. However, Stamp2 deficiency did not induce PASMC proliferation or migration (Fig. 4a, b). Furthermore, pulmonary MVEC apoptosis or viability were not affected by siRNA-mediated knockdown of Stamp2 (Fig. 4c, d, Supplementary Fig. 2), nor were IL-6 transcript levels (Supplementary Fig. 3). These results suggest that Stamp2 deficiency may impact pulmonary vascular remodeling via effects on other cell types than PASMC and EC.

### Stamp2 deficiency leads to increased pulmonary inflammation

**Fig. 5:**
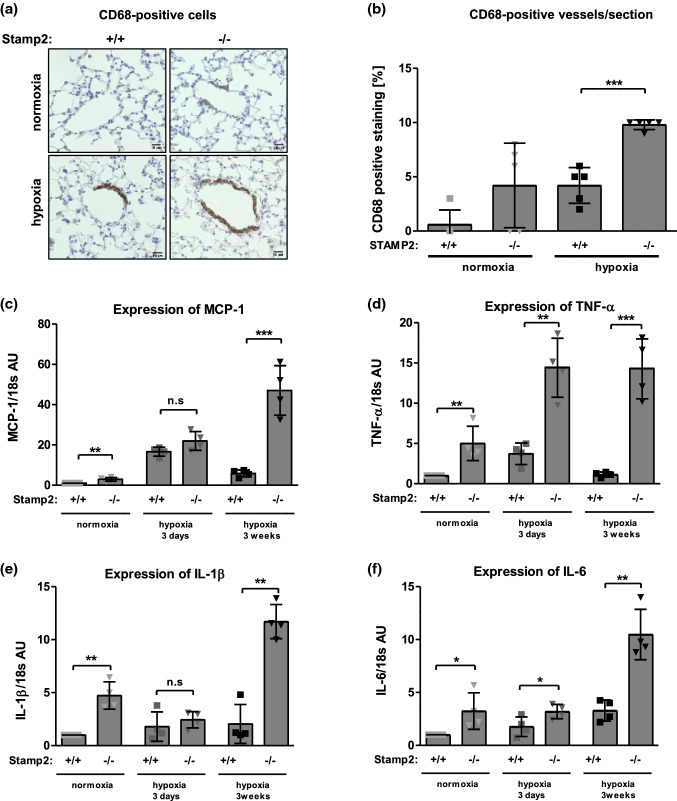
Stamp2 deficiency leads to increased pulmonary inflammation.** a** Representative immunohistochemical stainings demonstrating increased presence of CD68^+^ macrophages in the wall of small pulmonary vessels of normoxia- and hypoxia-exposed Stamp2-deficient mice versus WT controls (400 × magnification). **b** CD68^+^ vessels in percentage to the total number of vessels per defined area (60 × magnification) (*n* = 5,5,5,5). mRNA expression of **c** MCP-1, **d** TNF-α, **e** IL-1ß and **f** IL-6 in lung tissue from Stamp2-deficient and WT control mice after exposure to 0 days, 3 days or 3 weeks of hypoxia (*n* = 4,4,4,4). All data represent means ± SD of at least 4 independent experiments. *p < 0.05, **p < 0.01, ***p < 0.001 as assessed by two-tailed students *t*-test

Macrophages may indirectly influence pulmonary vascular remodeling processes. Moreover, macrophages seemed to be most susceptible to hypoxia-induced Stamp2 downregulation. Thus, we next focused on these cells and on inflammatory responses in the lung. Stamp2 deficiency led to enhanced infiltration of CD68^+^ macrophages at baseline (*p* = 0.08 vs. WT) which was further augmented under hypoxia (*p* < 0.001 vs. WT) (Fig. 5a, b). To further characterize the impact of Stamp2 deficiency on hypoxia-induced pulmonary inflammation, we investigated the expression of various PAH-associated cytokines in lung tissue. mRNA levels of MCP-1 as well as of TNF-α, IL-1ß, and IL-6 were robustly elevated in Stamp2-deficient mouse lungs as compared to WT under hypoxia at both 3 days and/or 3 weeks (Fig. 5c–f). In accordance with our earlier finding of enhanced NF-Kb signaling in Stamp2 deficiency [44], hypoxia led to increased expression of phosphorylated p65 in Stamp2-deficient lungs (Supplementary Fig. 4). Taken together, these data demonstrate that Stamp2 deficiency promotes pulmonary macrophage infiltration under hypoxic conditions and increased expression of inflammatory cytokines in the lung. The notion arises whether macrophages are implicated in the observed phenotype of aggravated pulmonary vascular remodeling via secretion of pro-inflammatory cytokines.

### Conditioned media of Stamp2-deficient macrophages augment PASMC proliferation and migration

**Fig. 6:**
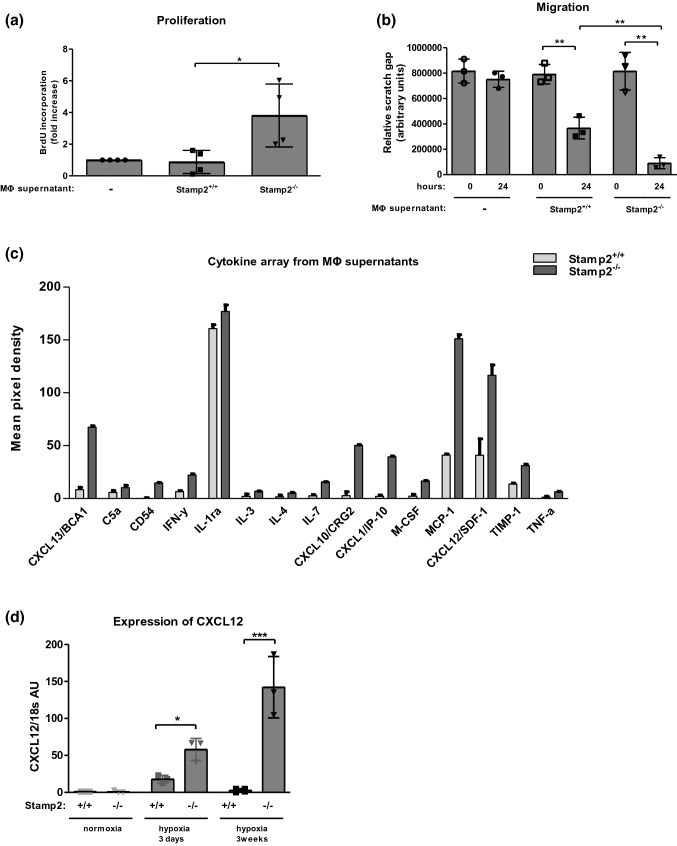
Macrophage supernatants of Stamp2-deficient mice promote PASMC proliferation and migration. **a** Proliferation (*n* = 4) and **b** migration of WT-PASMC in response to supernatants of primary, thioglycollate-elicited Stamp2-deficient or WT macrophages (MΦ) assessed by BrdU-incorporation and scratch assay (*n* = 3), respectively. Data are represented as means ± SD. *p < 0.05, **p < 0.01, as assessed by two-tailed students *t*-test. **c** Densitometric quantification of a cytokine array from Stamp2-deficient or WT MΦ supernatants (*n* = 2) **d** mRNA expression of CXCL12 in lung tissue from Stamp2-deficient and WT mice after exposure to 0 days, 3 days or 3 weeks of hypoxia (*n* = 3). All data represent means ± SD. *p < 0.05, **p < 0.01, ***p < 0.001 as assessed by two-tailed students *t*-test

To test this hypothesis, the impact of macrophage-derived secreted factors on cellular responses relevant to vascular remodeling were characterized. Conditioned media derived from activated primary macrophages were utilized to assess PASMC proliferation and migration. Conditioned media derived from Stamp2-deficient cells strongly induced PASMC proliferation (*p* < 0.05 vs. WT) (Fig. 6a). Furthermore, PASMC migration in response to macrophage supernatants was robustly augmented utilizing Stamp2^−/−^ mice as donors as compared to WT (*p* < 0.01) (Fig. 6b). To characterize potentially relevant secreted factors, a cytokine array was performed with supernatants of Stamp2-deficient and WT macrophages. Analyses revealed that several cytokines were differentially regulated in activated Stamp2^−/−^ and WT macrophages (Fig. 6c), including MCP-1 and CXCL12, that are known to induce PASMC proliferation and/or migration [11, 30, 41]. Therefore, these factors were selected for further validation experiments. To this end, mRNA expression levels were validated by qPCR. In line with the array data, mRNA expression of CXCL12 was strongly augmented in Stamp2 deficiency (*p* < 0.05/*p* < 0.001) (Fig. 6d), as were expression levels of MCP-1 and IL6 (Fig. 5).

### PASMC migration and proliferation in response to cytokines

**Fig. 7:**
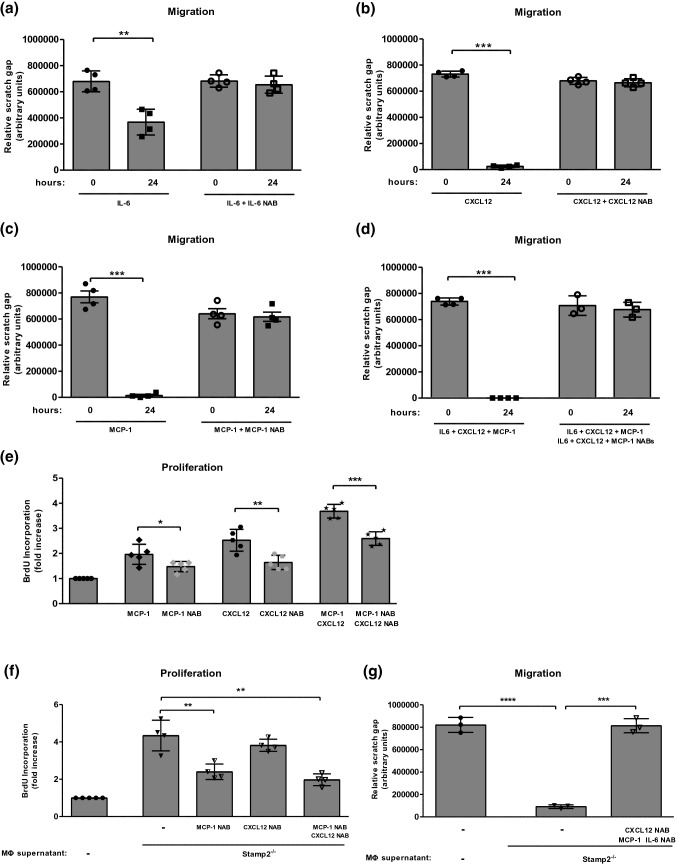
CXCL12, MCP-1 and IL-6 mediate cellular cross-talk between macrophages and PASMC. Migration (scratch assay) of WT-PASMC in response to **a** IL-6 (100 ng/ml) alone or in combination with an IL-6-neutralizing antibody (NAB) (*n* = 4), **b** CXCL12 (100 ng/ml) alone or with a CXCL12-NAB (*n* = 4) **c** MCP-1 (100 ng/ml) alone or with a MCP-1-NAB (*n* = 4,) **d** IL-6 (100 ng/ml) + CXCL12 (100 ng/ml) + MCP-1 (100 ng/ml) alone and in combination with their corresponding NABs (*n* = 4,4,3,3). **e** BrdU incorporation of PASMC in response to MCP-1 (100 ng/ml) and CXCL12 (100 ng/ml) alone and together with their corresponding NABs or with combined MCP-1 (100 ng/ml) and CXCL12 (100 ng/ml) either alone or with both NABs (*n* = 5). **f** BrdU incorporation (*n* = 5) of PASMC in response to macrophage supernatants of thioglycollate-elicited Stamp2-deficient primary macrophages in the absence and presence of MCP-1- or CXCL12-neutralizing antibodies (NAB). **g** Migration (scratch assay) of WT PASMC in response to supernatants of thioglycollate-elicited Stamp2-deficient primary macrophages (MΦ) in the absence or presence of NAB against CXCL12 + MCP-1 + IL-6 (*n* = 3). Data are represented as means ± SD. *p < 0.05, **p < 0.01, ***p < 0.001 as assessed by two-tailed students *t*-test

Whereas MCP-1, CXCL12 and IL-6 significantly induced PASMC migration (Fig. 7a–c), only MCP-1 and CXCL12 affected smooth muscle cell proliferation (Fig. 7e, supplementary Fig. 5), which is in line with previous publications [30, 40]. Additionally, the effect of combined incubation with cytokines was assessed. As expected, the observed effects were even stronger, with complete prevention by incubation with the respective combination of neutralizing antibodies (Fig. 7d, e).

To prove the pivotal role of CXCL12, IL-6 and MCP-1 in this scenario, PASMC were treated with supernatants of Stamp2^−/−^ macrophages as described above. Neutralizing antibodies against CXCL12, IL-6 and MCP-1 were applied to study the role of these specific cytokines. As shown in Fig. 7f, PASMC proliferation mediated by Stamp2^−/−^ macrophage supernatants was reduced by co-incubation with neutralizing antibodies directed against MCP-1 and CXCL12, and was almost completely blocked by incubation with both antibodies simultaneously. With regard to PASMC migration, the combination of neutralizing antibodies directed against CXCL12, MCP-1 and IL-6 completely abolished the effect of conditioned media derived from Stamp2-deficient macrophages (Fig. 7g). These data highlight the critical role of CXCL12, MCP-1 and IL-6 as mediators of the cross-talk between macrophages and smooth muscle cells in Stamp2 deficiency.

## Discussion

In this study, we show that Stamp2 deficiency results in an aggravated inflammatory response in the lung and in worsening of hypoxia-induced pulmonary vascular remodeling and pulmonary hypertension in vivo. In the absence of Stamp2, secreted factors from macrophages and inter-cellular cross-talk promotes proliferation and migration of smooth muscle cells, and these effects were abolished by neutralizing antibodies. As macrophages were significantly more abundant in Stamp2-deficient lungs under hypoxia, this mechanism appears to be critically involved in the observed phenotype. Importantly, Stamp2 expression is substantially decreased in both experimental and human PAH. Thus, these data suggest that loss of Stamp2 expression in PAH might be a general driving factor during disease progression. Together with our earlier work [44], the present study puts Stamp2 in a critical position as a regulator of macrophage inflammation, playing a central role in cardiovascular diseases such as atherosclerosis, diabetes and pulmonary hypertension.

Although statistically significant, the effects of Stamp2 deficiency on pulmonary vascular remodeling and pressure are modest, providing an explanation for the lack of difference in RV hypertrophy between genotypes. These results suggest that pro-inflammatory mechanisms alone are not sufficient to cause severe PAH. Nevertheless, in our opinion the observed effects are highly relevant, as the loss of mechanisms protecting from overt inflammation is particularly important if disease-promoting mechanisms are enhanced at the same time (which is the case in all forms of PAH). Thus, our data supports the concept that an imbalance between drivers of disease and preventive factors (such as Stamp2) is a key feature for PAH progression.

Stamp2 serves critical functions at the interface of metabolic and inflammatory pathways through actions in target cells critical for metabolic and immune regulation. Based on published literature so far, this applies to adipocytes [51], hepatocytes [24] and macrophages [44]. Here, we also detected significant expression of Stamp2 in endothelial cells and suspected a function of the protein in these cells in pulmonary remodeling. However, we could not find any relevant cellular response (including expression of the inflammatory cytokine IL-6) from endothelial cells that was different in Stamp2 knock-down. We previously showed that—despite of profound effects on atherogenesis—Stamp2 deficiency did not cause impaired systemic glucose tolerance [44], pointing towards a more direct role of Stamp2 in the control of cellular inflammatory responses. Here, we show that, under hypoxic stress, Stamp2 deficiency directly caused increased inflammatory output from macrophages. Stamp2′s role in the control of inflammatory responses appears to be confined to inflammatory cells, whereas Stamp2 in endothelial cells might serve other functions that remain to be elucidated. Importantly, secreted factors from macrophages induce smooth muscle cell proliferation and migration, both critical events during vascular remodeling in PAH. By means of a cytokine array and validation experiments, we identified three cytokines that are most relevant to pulmonary vascular remodeling, MCP-1, IL-6 and CXCL12, that were robustly upregulated and secreted by Stamp2-deficient macrophages. All of these were shown to be regulated by the transcription factor NF-KB [15, 35] that was demonstrated to be activated in Stamp2 deficiency. The mechanism behind this is the trapping of the negative NF-KB regulator NmrA-like family domain-containing protein 1 (Nmral) in the cytosol, thereby promoting enhanced transcription of NF-KB target genes [44].

Secreted MCP-1, IL-6 and CXCL12 were shown to impact cellular responses in smooth muscle cells that are potentially relevant during vascular remodeling processes. MCP-1 [41, 43] and CXCL12 [4, 11, 23] impact both smooth muscle cell proliferation and migration, whereas IL-6 appears to preferentially induce smooth muscle cell migration [30, 40], findings that were confirmed in our analyses. Thus, these cytokines were the most promising candidates potentially explaining the observed inter-cellular cross-talk between macrophages and smooth muscle cells. Utilizing neutralizing antibodies, we could indeed demonstrate that all three cytokines are critically involved in the observed phenotype. This was highlighted by the finding that the combination of neutralizing antibodies abolished both smooth muscle proliferation and migration induced by Stamp2-deficient macrophage supernatants.

Importantly, Stamp2 expression is robustly decreased in experimental and human PAH, most probably due to increased levels of reactive oxygen species (ROS) [27] that are present in PAH and in hypoxia [6]. Based on these results, macrophage Stamp2 arises as a powerful anti-inflammatory player that controls critical cellular responses in neighboring cells in PAH. Thus, Stamp2 may represent a suitable target for pharmacological intervention. Indeed, animal studies utilizing adenoviral constructs to induce overexpression of Stamp2 in the liver were effective to improve insulin resistance and hepatic steatosis in response to high-fat diet [24] or in diabetic mouse models [49]. The same appears to be true for experimental atherosclerosis in mice [50]. Whereas these data strengthen the critical role of Stamp2, such strategies cannot be applied in humans. Recently, a high-throughput screening identified the “selective” phosphodiesterase-3 inhibitor cilostazol as a Stamp2 enhancer that increases hepatic Stamp2 expression via AMP-activated protein kinase (AMPK) in vitro and in vivo [32]. This led to decreased lipid accumulation in a mouse model of non-alcoholic fatty liver disease. Cilostazol is approved as a second-line agent for peripheral artery disease. Consequently, restoration of protective mechanisms such as Stamp2 in addition to targeting disease-promoting pathways may evolve as a feasible and powerful strategy to improve treatment options in PAH and potentially other conditions.

In summary, we identify a novel modulator of pulmonary vascular remodeling and PAH that represents a suitable target for pharmacological intervention—potentially utilizing cilostazol as it is a readily available agent. Future studies will analyze the effects of this drug on experimental pulmonary hypertension and atherosclerosis, and, if successful, also in human disease.

## Electronic supplementary material

Below is the link to the electronic supplementary material.

Supplementary file 1 (PDF 210 kb)
