# Supplementary material for: The six-transmembrane protein Stamp2 ameliorates pulmonary vascular remodeling and pulmonary hypertension in mice
Source: Basic Res Cardiol. 2020 Nov 13;115(6):68. doi: 10.1007/s00395-020-00826-8 (PMC7666299; doi:10.1007/s00395-020-00826-8)
Supplement: Supplementary file 1 — (PDF 210 kb) [file 395_2020_826_MOESM1_ESM.pdf]

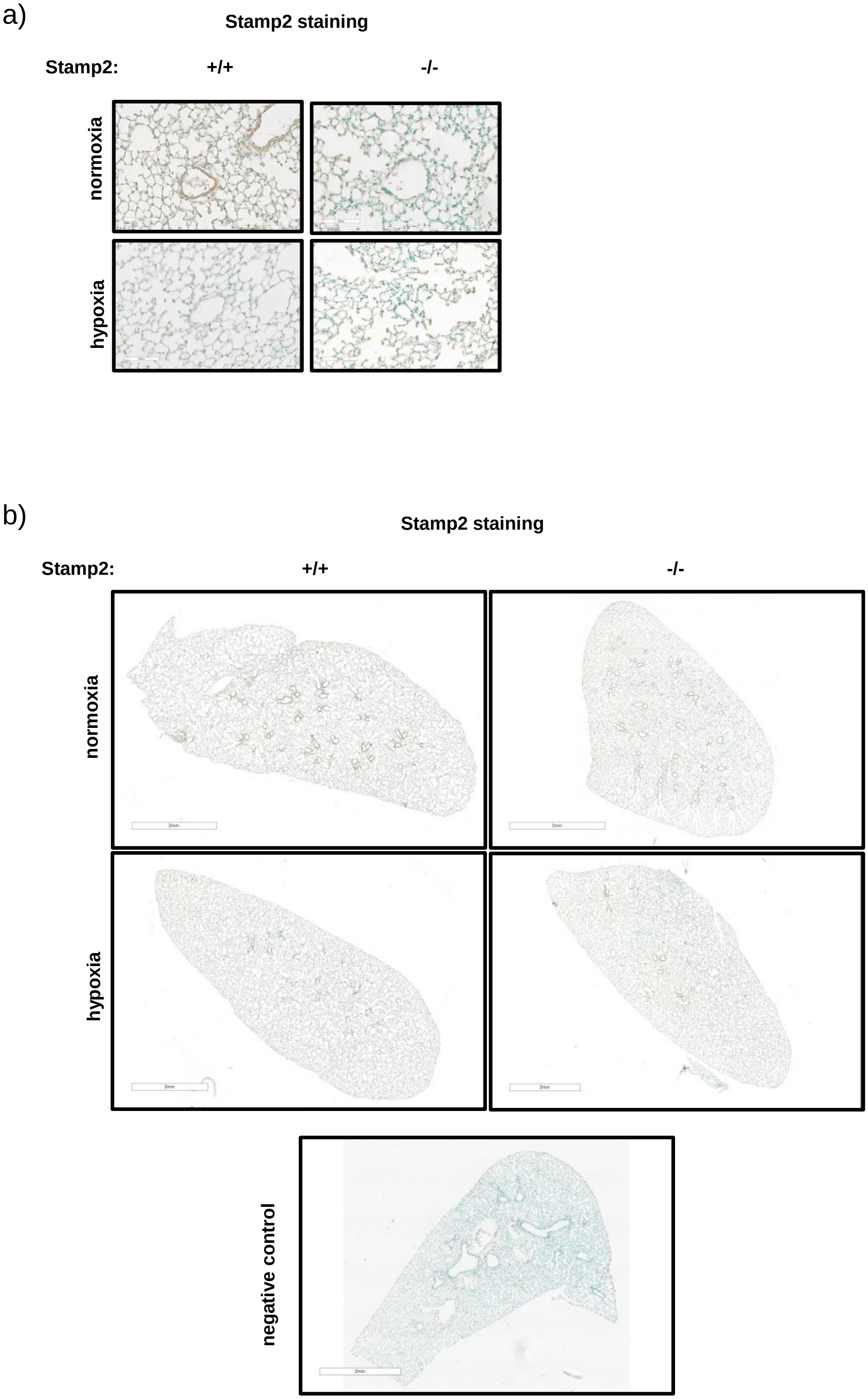

Supplementary Figure 1

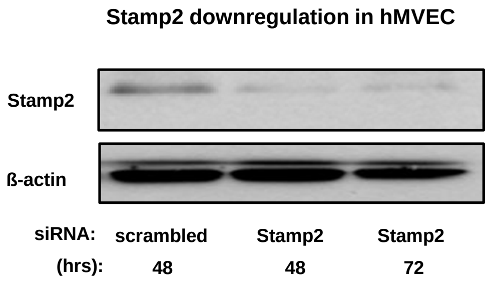

Supplementary Figure 2

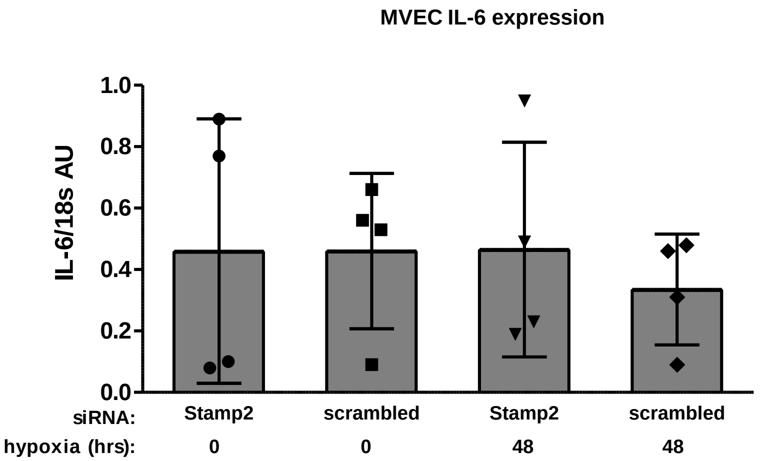

Supplementary Figure 3

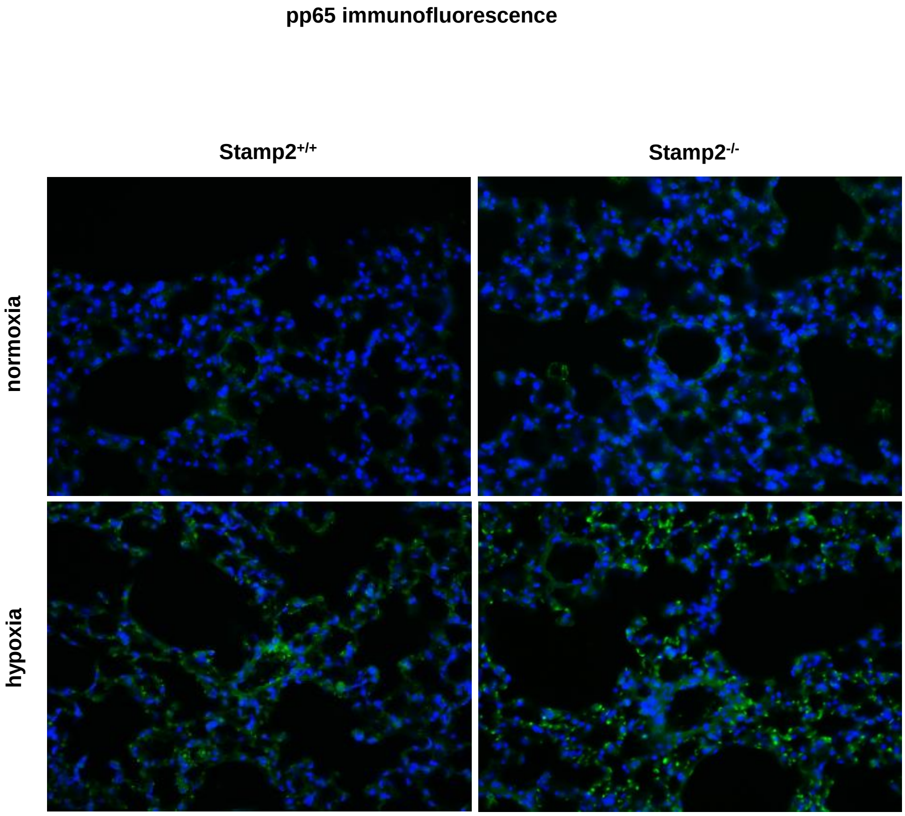

Supplementary Figure 4

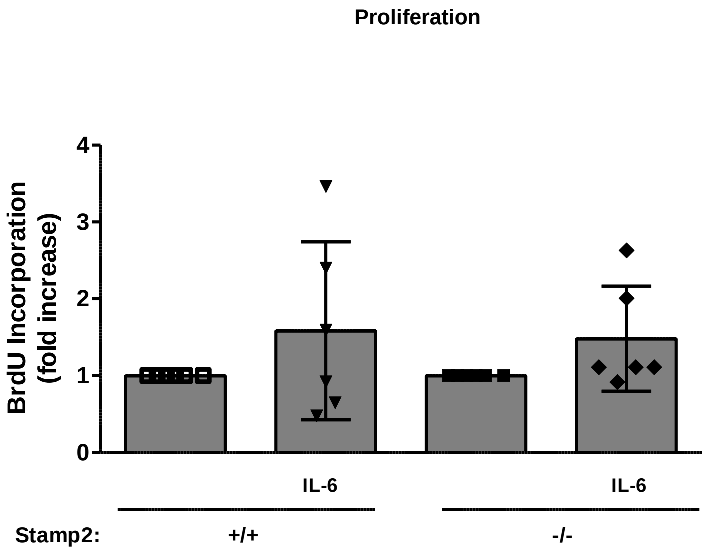

Supplementary Figure 5
